# Development and qualification of clinical grade decellularized and cryopreserved human esophagi

**DOI:** 10.1038/s41598-023-45610-5

**Published:** 2023-10-25

**Authors:** William Godefroy, Lionel Faivre, Caroline Sansac, Briac Thierry, Jean-Marc Allain, Patrick Bruneval, Rémy Agniel, Sabrina Kellouche, Olivier Monasson, Elisa Peroni, Mohamed Jarraya, Niclas Setterblad, Massymissa Braik, Benjamin Even, Sophie Cheverry, Thomas Domet, Patricia Albanese, Jérôme Larghero, Pierre Cattan, Lousineh Arakelian

**Affiliations:** 1 Service de Chirurgie Viscérale, Cancérologique et Endocrinienne, Hôpital Saint-Louis - Université Paris Cité, Paris, France; 2 Unité de Thérapie Cellulaire, Hôpital Saint-Louis, AP-HP, Paris, France; 3 CIC de Biothérapies CBT 501, Paris, France; 4 Human Immunology, Pathophysiology, Immunotherapy / HIPI / INSERM UMR976, Laboratoire de Biotechnologies de Cellules Souches, Université Paris Cité, 75010 Paris, France; 5 Banque de Tissus Humains, Hôpital St-Louis, AP-HP, Paris, France; 6 Service d’ORL Pédiatrique, AP-HP, Hôpital Universitaire Necker, 75015 Paris, France; 7 LMS, CNRS, Ecole Polytechnique, Institut Polytechnique de Paris, Palaiseau, France; 8 Inria, Paris, France; 9 Service d’Anatomie Pathologie, Hôpital Européen Georges Pompidou, AP‐HP, Paris, France; 10 Equipe de Recherche sur les Relations Matrice Extracellulaire-Cellules, ERRMECe (EA1391), Institut des Matériaux, I-MAT (FD4122), CY Cergy Paris Université, Cergy-Pontoise, France; 11 CNRS, BioCIS, CY Cergy Paris Université, 95000 Cergy Pontoise, France; 12 CNRS, BioCIS, Université Paris-Saclay, 92290 Châtenay-Malabry, France; 13 UMS Saint-Louis US53 / UAR2030, Institut de Recherche Saint-Louis Plateforme Technologique Centre, Université Paris Cité - Inserm - CNRS, Paris, France; 14 Laboratoire Gly-CRRET, Université Paris Est Créteil, Université Paris Est, EA 4397 ERL CNRS 9215, Créteil, France; 15 Centre MEARY de Thérapie Cellulaire Et Génique, AP-HP, Hôpital Saint-Louis, 75010 Paris, France

**Keywords:** Biomaterials, Tissues

## Abstract

Tissue engineering is a promising alternative to current full thickness circumferential esophageal replacement methods. The aim of our study was to develop a clinical grade Decellularized Human Esophagus (DHE) for future clinical applications. After decontamination, human esophagi from deceased donors were placed in a bioreactor and decellularized with sodium dodecyl sulfate (SDS) and ethylendiaminetetraacetic acid (EDTA) for 3 days. The esophagi were then rinsed in sterile water and SDS was eliminated by filtration on an activated charcoal cartridge for 3 days. DNA was removed by a 3-hour incubation with DNase. A cryopreservation protocol was evaluated at the end of the process to create a DHE cryobank. The decellularization was efficient as no cells and nuclei were observed in the DHE. Sterility of the esophagi was obtained at the end of the process. The general structure of the DHE was preserved according to immunohistochemical and scanning electron microscopy images. SDS was efficiently removed, confirmed by a colorimetric dosage, lack of cytotoxicity on Balb/3T3 cells and mesenchymal stromal cell long term culture. Furthermore, DHE did not induce lymphocyte proliferation in-vitro. The cryopreservation protocol was safe and did not affect the tissue, preserving the biomechanical properties of the DHE. Our decellularization protocol allowed to develop the first clinical grade human decellularized and cryopreserved esophagus.

## Introduction

Tissue Engineering (TE) is a promising and credible alternative to current esophageal replacement methods by gastroplasty or coloplasty, allowing the preservation of intra-abdominal organs and of the healthy part of the esophagus by replacing only the pathologic segment^1,2^. For this purpose, it is necessary to develop a biocompatible scaffold, leading to cell colonization and tissue repair after in vivo implantation^3^. The ideal substitute should be sterile, non-immunogenic, and have biomechanical properties similar to those of the organ to be replaced^4^.

Many strategies have been developed for alternative full thickness circumferential esophageal replacements, with different natural or synthetic materials^1,2,5–7^. Unfortunately, the majority of these materials have shown disappointing results regarding their biocompatibility and their capacity to promote tissue regeneration^5,7^. To date, none of them has led to satisfactory preclinical or clinical results.

Organ decellularization has become a promising TE strategy for organ replacement. The purpose of this method is to remove cells and their genetic material in order to reduce inflammation and immune reaction, while preserving the components of the extracellular matrix (ECM) and its complex macro and micro architecture^3^. In order to validate a full decellularization, Crapo et al*.* suggested a few criteria including a complete removal of cells and nuclei, evaluated by histology and after DAPI staining, reducing DNA to a quantity inferior to 50 ng/mg of dry mass, and dsDNA fragment size inferior to 200 bp^8^.

Indeed, it has been shown that organ specific ECMs have innate properties for the regeneration and repair of a given organ, by allowing a compartment specific recolonization of cells in vivo^9,10^*.* As for the esophagus, a number of teams have proposed decellularization^11–14^ respecting recommendations of Crapo et al*.*^8^ but none of them obtained a decellularized esophagus of clinical grade, i.e. respecting criteria of the European guidelines for "cell and tissue products", transferrable to a GMP structure for a clinical use. In a previous study, we developed and validated our own clinical grade decellularization protocol in a porcine model^15^.

This decellularized porcine esophagus (DPE) was then implanted in vivo by Levenson et al.^16^ for a 5-cm long circumferential full thickness replacement of the thoracic esophagus in pigs, under the cover of a temporary esophageal endoprothesis. The procedure was associated with the realization of an epiplooplasty around the DPE. Analysis of the graft area after sequential sacrifices of the animals showed that the DPE was successfully revascularized after two weeks, and a full mature epithelium was observed in the DPE after three months. Beginning of muscle regeneration appeared from 2 months. Pigs conserved their nutritional autonomy after surgery, with a median follow-up of 112 days. A recurrent problem was graft area stenosis mainly due to recurrent early stent migration, which was solved by optimizing stent fixation procedure. These promising results led us to adopt this strategy for future human applications.

One of the main issues with DPE is its animal origin and the regulatory constraints related to it. Porcine tissue graft could induce an acute or chronic rejection due to the presence of antigens such as alpha-galactosyl epitopes that are absent in humans^17^. Furthermore, questions may raise concerning the transmission of porcine pathogens to humans^18^.

Thus, we decided to adapt our protocol to develop a decellularized human esophagus (DHE) to pave the way for future clinical applications.

The main objectives of our study were evaluating the feasibility and validating a clinical grade decellularization protocol for human esophagus, as well as developing a cryopreservation protocol for a ready to use DHE bank.

## Material and methods

### Esophageal retrieval

**Figure 1:**
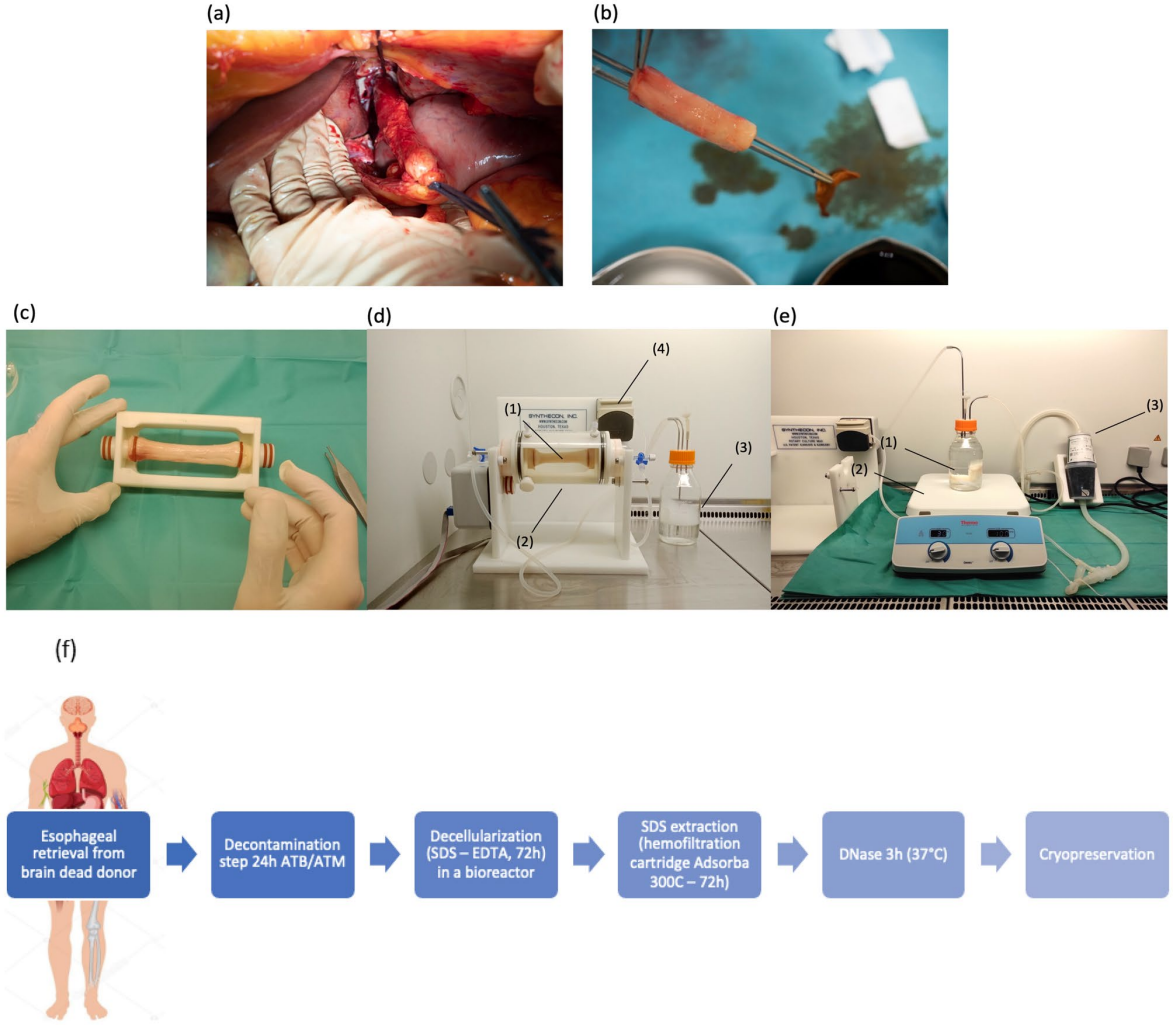
Harvesting and preparation of the retrieved esophagus during brain dead donor harvest procedure. (**a**) Shows the retrieval by transhiatal approach, the esophagus is ligated above and beyond and resected. It is rinsed, decontaminated outside and inside the lumen (**b**) after periesophageal tissues were removed. Native esophagus on the bioreactor support before decellularization (**c**). Esophagus inside the bioreactor during the process (**d**). The esophagus (d1) is placed inside the rotating cylinder filled with the SDS/EDTA solution (d2). A bottle containing the same solution (d3) connected to a peristaltic pump (d4) functioning as a closed circuit allows irrigation of the esophageal lumen. Method of SDS elimination after decellularization with the activated charcoal cartridge (**e**). The DHE is placed in a clean bottle containing physiological serum 0.9% (e1) over a magnetic agitator at 30°C (e2) and the solution is filtered by the cartridge (e3) through the closed circuit trained by the peristaltic pump. The final decellularization process with SDS extraction using the activated charcoal cartridge is resumed in (**f**).

Esophageal grafts were harvested from human deceased donors following a brain-death or Maastricht category III circulatory arrest. Non-opposition, informed consent was obtained from the donors’ families for research purposes. The graft retrieval authorization was granted by the "Agence de la Biomédecine", grant number PFS18-018 in Saint-Louis Hospital, Université Paris Cité, France. Under sterile conditions, 15 cm of thoracic esophagi were removed either by thoracotomy or by transhiatal approach in case of only intra-abdominal organ retrieval. The esophageal segment was harvested between two clamps so that there was a minimum digestive contamination (Fig. 1a,b). Esophageal retrieval was the last performed, in order to avoid contamination of the other grafts.

### Decellularization of human esophagi

The protocol was adapted from the one previously developed by our team in a porcine model^15^. It consisted of five major steps: decontamination, decellularization with a detergent treatment to remove cells, rinsing and removal of the residual detergent, incubation with DNase followed by a rinsing step before preservation. All these steps were carried out under sterile condition in a laminar flow hood.

#### Esophageal decontamination

Immediately after retrieval in the operation room, esophagi were cleaned with Povidone-Iodine (Betadine^®^ dermique 10%, Meda Pharma, Paris, France) and rinsed with an antibiotic and antimycotic (ATB/ATM) solution composed of physiologic serum 500mL, Gentamicin 160 mg (Panpharma, Boulogne Billancourt, France), Clindamycin 300 mg (Fresenius Kabi, Bad Homburg vor der Höhe, Germany), Vancomycin 250 mg (Canonsburg, PA, USA) and 50 mg of Amphotericin B (Fungizone^®^, Bristol-Myers Squibb, New-York, USA). Twenty esophagi were then transported to our laboratory in the same ATB/ATM solution at 4 °C. At reception, the solution was renewed and the esophagi were decontaminated at 25 °C, under an agitation of 225 rpm for 24 h. The objective was to remove most of the microbial contamination before the decellularization.

#### Decellularization protocol

After the decontamination step, the esophagi were rinsed 3 times in sterile water for 1h under agitation to remove the residual ATB/ATM. The esophagi were then placed in a bioreactor (Synthecon, Texas, USA) a rotating perfusion system that facilitates decellularization by mechanical action. The procedure was performed in sterile conditions under a laminar flow hood. The bioreactor was composed of a support for the esophagus (Fig. 1c) placed in a rotating glass cylinder, allowing a continuous unidirectional flow irrigating the lumen of the graft. This flow inside the lumen was generated with a 500 mL bottle connected in series to a peristaltic pump and the cylinder. The cylinder and the bottle were filled with a solution of 2% Sodium Dodecyl Sulfate (SDS, Euromedex, France) and 5 mM Ethylene Diamine Tetraacetic Acid (EDTA, Euromedex, France). The SDS/EDTA solution was renewed after 24 h and the esophagus was treated for a total of 72 h under constant rotation (27 rpm) and flow inside the lumen at 27 mL/min (Fig. 1d).

Treated esophagi were then rinsed inside the cylinder with sterile water for four cycles of one hour and one last cycle of two hours with the same parameters of flow and rotation.

To remove the residual SDS, esophagi were then incubated with an ion-exchange resin Amberlite^®^ XAD16N (Sigma, France) under agitation at 25 °C, 225 rpm, in an orbital agitator (Intelli‐mixer, ELMI, Riga, Latvia).

To remove DNA, DHE were incubated for 3h at 37 °C and 4 rpm on the rotating agitator with 10 U/mL of DNase (Pulmozyme^®^, Roche, Boulogne‐Billancourt, France) in 22.5 mL of Phosphate Buffer Saline (PBS) with calcium and magnesium (Eurobio, Courtaboeuf, France). The DNase was then rinsed in PBS/EDTA and DHE were then stored at 4 °C in PBS, before further characterization.

#### Optimization of the SDS-removal method

In order to optimize SDS removal step for a clinical grade application, ion-exchange resin was replaced by an activated charcoal cartridge, commonly used for toxic elimination in intensive care units. The SDS released from the DHE is adsorbed by the cellulose-coated activated charcoal.

After SDS treatment and initial rinsing steps, the esophagi were placed in a 500 mL bottle filled with sterile water and connected in series to the Adsorba 300C (Baxter, USA) cartridge forming a closed circuit with a peristaltic pump (27 mL/min). The bottle was placed on a warming magnetic agitator Cimarec™ (Thermo Fisher Scientific Inc., Waltham, USA) at 30 °C and 100 rpm and the solution was continuously filtered for 72 h (Fig. 1e). The final protocol is resumed in the Fig. 1f.

### Characterization of the DHE

#### Microbiological analysis

Sterility was evaluated on samples of esophagi before and after ATB/ATM treatment and at the end of the whole process. Samples were incubated in Schaedler broth (Biomérieux SA, Craponne, France) for 10 days and then seeded into Chocolate agar + PolyViteX (Biomérieux SA) for aerobic culture, Columbia agar + 5% sheep blood (Biomérieux SA) for anaerobic culture, and Sabouraud chloramphenicol gentamicin agar (Bio‐Rad Inc, Hercules, USA) for fungal culture. The bacterial media were incubated at 37 °C for 8 days and the fungal medium at 30 °C for 11 days.

#### Histology, elastin quantification and scanning electron microscopy

Analysis of the general structure and residual cells was carried out both on native and decellularized esophagi. Samples were fixed in 4% buffered Formalin (PFA, Alfa Aesar™, Thermofisher, Germany) then embedded in paraffin. Five micrometers-thick sections were stained with hematoxylin–eosin–saffron (HES) for the general structure and residual cells, picrosirius red for the collagen and orcein for the elastic fibers. Images were obtained with a LEICA DM6 microscope.

Elastin in native esophagus and DHE (n = 2), was quantified using Fastin™—Elastin assay kit (Biocolor life science assays, UK), according to the manufacturer’s instructions. The mucosa/submucosa and the muscle/adventice were mechanically separated using forceps. Humidity was withdrawn by pressing samples in a sterile compress. They were then cut into small pieces weighing between 30 and 80 mg. Elastin was solubilized from the tissue in 750 µl of 0.25 M oxalic acid, at 100 °C for one hour. Two consecutive extractions per sample were performed. The extracted elastin was precipitated and centrifuged at 13000 g. It was then stained with Dye Reagent for one hour, centrifuged, and the supernatant was eliminated. Elastin bound dye was then solubilized in Dye Dissociation reagent. In parallel to the samples, known concentrations of elastin standard, ranging from 0 to 750 µg/ml were used to establish the standard curve.

To calculate the total elastin concentration in the tissue, the sum of the two extractions was divided by the weight of the samples. The final concentration was represented as µg of elastin/mg of wet tissue. Results were then compared with an unpaired t-test, using Prism software (GraphPad).

To perform the scanning electron microscopy (SEM) analysis, circumferential segments of native and decellularized esophagi were fixed in glutaraldehyde 2% (Sigma) diluted in cacodylate buffer 0.1 M (Alfa Aesar™, Thermofischer, Germany), pH 7.3 at 4 °C for 24 h. Samples were placed without any drying/metallization onto a cool stage (Deben) and cooled down to − 25 °C. SEM micrographs were acquired with a Scanning Electron Microscope Gemini SEM 300 (Zeiss) operating in variable pressure mode (60 Pa) at 10 keV.

#### DNA quantification and electrophoresis on agarose gel

To evaluate the efficiency of the decellularization, DNA extraction and quantification were performed. Samples of DHE before and after DNase treatment were lyophilized, weighed and digested overnight by proteinase K at 56 °C. The DNA was extracted with DNeasy Blood and Tissue kit (Qiagen, Hilden, Germany) according to the manufacturer’s protocol. Extracted DNA was quantified by Nanodrop (Thermofisher) and the results were expressed as ng/mg of dry tissue. The DNA fragments' length was evaluated after electrophoresis for 1h at 150 V in a 2% agarose gel with SYBR^®^ safe (Thermofisher) and analyzed with iBright1500 (Invitrogen). Image acquisition was done on auto-exposure mode, for an exposure time of 23 ms.

#### Glycosaminoglycans (GAG) purification and quantification

Total sulfated glycosaminoglycans (GAGs) were extracted from dried/lyophilized native and DHE samples and purified as previously described^19^. Total sulfated GAGs were quantified using 1,9 dimethylmethylene blue procedure (DMMB, Sigma Aldrich, Saint Louis, USA). Absorbance was read at 656 nm on a Spark^®^ multimodal plate reader (TECAN, Switzerland) giving the optical density (OD) for each well.

### GAG immunostaining

Immunostaining of the GAGs was performed as previously described^20^. Slides of native and decellularized esophagus were incubated for 10 min with 50‐mM NH4Cl and saturated with 3% bovine serum albumin/PBS for 45 min. GAGs were then stained overnight at 4°°C with phage display antibodies linked to VSV (vesicular stomatitis virus) sequences: EV3C3V (1:5) binds to heparan sulfate (HS) and LKN1 (1:5) binds to dermatan sulfate (DS), an epimerized form of chondroitin sulfate (CS) also named CS‐B. All dilutions were performed in PBS 1X / bovine serum albumin 3%. Bound antibodies were detected with rabbit anti‐VSV polyclonal antibody followed by incubation with a goat anti‐rabbit antibody coupled to FITC. Controls of immunostaining were obtained with anti‐VSV and goat anti‐rabbit FITC staining without primary antibodies. The slides were also stained with 4′,6‐diamidino‐2‐phenylindole (DAPI) to observe cell nuclei.

Fluorescent intensity of the images was acquired with the LSM 800 microscope (Zeiss, Oberkochen, Germany) and analyzed with the software ZEN Blue edition V 2.1.

### Heparin/GAG ELISA competition assay towards heparin binding proteins (HBP)

GAG abilities to interact with HBP were evaluated by an ELISA based competition assay as described^21^. Briefly, 96- wells ELISA plates (Costar) are coated with heparin-BSA conjugate and optimized doses of Recombinant Human HBP (FGF-2; VEGF) were added to each well, together with increasing concentration of extracted GAG. This allows to quantify the abilities of soluble GAG tested to interact with HBP and compete with immobilized-heparin. After washing steps, HBP remaining bond to coated heparin-BSA on plate are quantified by specific antibody sandwiches according to classical ELISA detection^21^. The maximal binding (100%) was determined in presence of the HBP and in absence of extracted GAG. Inhibitory Competitive dose (IC50) was defined as GAG concentration (µg/ml) that inhibit 50% of the HPB binding to immobilized heparin. IC50 value obtained for Heparin positive control samples was used to define a relative affinity of 100% and to report % of affinities of other GAG. For instance, a tenfold higher IC50 value of GAG sample “x” as compared to GAG sample “y” for an HBP corresponds to a tenfold lower affinity of GAG “x” as compared to GAG “y”, since tenfold higher concentration of “x” than “y” are necessary to compete for HBP binding to heparin.

#### In-vitro biocompatibility assays

##### Cytotoxicity analyses on Balb/3T3 cells

The cytotoxicity of the DHE was evaluated according to the European Standard ISO 10993-5-2009^22^. Balb/3T3 clone A31 cells (ATCC, U.S.A) were cultured in Dulbecco’s modified eagle medium (DMEM) High Glucose 4,5 g/L (ATCC, U.S.A.) supplemented with 10% bovine calf serum (BCS, ATCC) and 1% ATB/ATM. Cells were seeded at 15 000 cells/cm^2^ in a 48 wells plate and incubated at 37°C for 24 h. DHE samples of 5 mm diameter were prepared with a biopsy puncher (Kai medical, Solingen, Germany) and incubated with 200 µL/well of extraction medium (same composition as the Balb/3T3 culture medium but supplemented with 2% BCS). At 24 h, the supernatant was used to replace the culture medium of the Balb/3T3 cells and culture medium of the control cells was renewed. Cells were cultured for 72 h and then detached by a trypsin treatment. Viability was evaluated by Annexin V-FITC and 7AAD staining (Beckman Coulter France, Villepinte, France) according to the manufacturer’s instructions. Results were analyzed by flow cytometry using the Attune™^−^NxT cytometer (ThermoFiscer scientific).

##### Lymphocyte proliferation assay

To assess the in vitro immunogenicity of the DHE, human peripheral blood mononuclear cells (hPBMCs), were isolated on Ficoll gradient and stained with carboxyfluorescein succinimidyl ester fluorescent 5 μM (CFSE, Life Technologies, Carlsbad, USA) according to the manufacturer’s instructions. Samples of 5 mm diameter of DHE were placed in a 96 wells plate. PBMCs were seeded at 10^4^ cells/well in a culture medium composed of Roswell Park Memorial Institute glutamax (RPMI, Gibco^®^ Waltham, MA, USA) supplemented with 10% FBS and 1% ATB/ATM. PBMCs were seeded alone, in contact with fragments of DHE or with 5 μg/mL of phytohemagglutinin‐L (PHA-L, Roche) as a positive control. After 5 days, cell proliferation was analyzed by measuring the fluorescence intensity decrease by flow cytometry with the Attune™^−^NxT (Thermofisher, USA). The percentage of division (PD) was calculated by using the formula:

PD = divided events/(divided events + undivided events).

##### F of the DHE with human mesenchymal stromal cells (hMSCs)

In order to perform a biocompatibility test of the DHE and to show its ability to support cell attachment, lack of cytotoxicity, migration and proliferation, we seeded bone marrow hMSCs at passage 2 on samples of DHE. The hMSCs were isolated and characterized as previously described^15,23^.

Segments of 2 cm of three DHE were prepared and cultured in a 50 mL tube Cellstar^®^ CELLreactor™ (Greiner bio one) filled with a suspension of 5.10^6^ cells in 30 mL of MSC culture medium (Fig. 5c). Samples were placed on a rotating agitator (Intelli‐mixer, ELMI, Latvia) at 1 rpm and 37 °C. Medium culture was renewed after 24 h to remove unattached cells and then every 72 h. Samples were cultured for 14 days, then fixed with PFA 4% and analyzed by HES coloration.

#### Qualitative evaluation of metabolic activity by colorimetric test after DHE seeding

Qualitative evaluation of metabolic activity after cell seeding on DHE were analyzed using CellTiter 96^®^ AQueous Non-Radioactive Cell Proliferation Assay (MTS, Promega, USA). About one cm^2^ of DHE (3 before and 3 after cryopreservation) after 14 days of culture with hMSC were incubated at 37 °C, 5% CO_2_ for 1 h into 600 µL culture medium with 1% of MTS solution. Control group consist to DHE without cell. The purple color observed in the DHEs correspond to the presence of living cells.

#### hMSC quantification after DHE seeding

hMSC quantification was performed on six samples of DHE (3 before and 3 after cryopreservation) after 14 days of culture with hMSC. About one cm^2^ of DHE seeded with hMSC was incubated during 5 min with propidium iodide at 30 µg/ml (Sigma Aldrich) labelling nucleus of dead cells then incubated during 5 min with Hoechst 33,345 at 10 µg/ml (Sigma Aldrich) labelling nucleus of all cells. After several PBS washings, four images were acquired at four locations using a fluorescence microscope (Spinning disc, Nikon). All images are a Z-stack projection of 10 to 20 sections spaced from 3 µm on thickness of 30 to 60 µm. Images were processed using the software Image J to count viable cells and determine hMSC concentration per mm^3^ of DHE.

##### Residual SDS quantification

The concentration of residual SDS was measured with a spectrophotometric method, based on the use of a carbocyanine dye (Stains-all) the color of which changes from intense fuchsia to yellow upon addition of SDS.

##### Buffers and reagent solutions

Stains-all was dissolved in N, N-dimethylformamide to give a stock solution of 2.0 mg/ml. Working solutions were diluted 1:20 with MilliQ water into dark amber Falcon™ tube and stored in the dark at 2–8°C.

##### Standards and sample preparation

SDS standard concentrations were prepared by dilution of the 1% stock solution with PBS. Six SDS standard solutions were prepared, ranging from 0.01 to 0.00025% SDS and stored at room temperature. The linearity of the curve representing absorbance versus concentration was validated prior to the dosage.

Samples of DHE were lyophilized and stored at 4°°C. Pieces up to 30 mg were weighed and placed into 2 mL tubes with 1 mL of PBS. Samples were grinded with a Tissue Lyser (Qiagen) at 50 rpm for 15 min and placed in an incubator for 48 h at 37°C. Supernatants were collected and analyzed for the presence of SDS.

The concentration of the SDS were also quantified in the PBS in which DHE were preserved after decellularization (500 mL/5cm long DHE).

##### Spectrophotometric analysis of detergent concentration

All microtiter assays were performed in a Dulbecco’s PBS buffer.

PBS buffer (210 µL/well) was dispensed using a multichannel pipet. 15 µl of each standard, or sample was dispensed into the well of a 96 wells "ultra-low UV" (Corning). A multichannel pipet was then used to dispense 75 µl/well of Stains-all working solution. The microtiter plate was immediately placed in the incubator/reader and shaken for 20 s, allowed to incubate unshaken for 20 s, and then read for absorbance using a 450 nm filter with a microplate reader "Varioskan Lux" (Thermo Scientific). Results were then standardized as μg of SDS/mg of dry DHE.

### Biomechanical properties

Biomechanical properties of DHE using uniaxial traction assays as previously described^15^ were performed. Two frozen/thawed and 2 unfrozen DHE were used: DHE were cut into approximately 1 cm wide and 3 cm long strips, in the longitudinal and in the transverse directions.

Prior to the experiment, the dimensions of each sample were measured using a caliper. The tensile tests were performed at a strain rate around 0.6% s^−1^, continuously, until the rupture of the sample. During each test, the displacement of the grips and the force were recorded every second. Images were also acquired to verify that no slipping occurred during the assay.

The force was divided by the initial section of the sample to obtain the nominal stress. Stretch was determined through the machine displacement, divided by the initial sample length. Four parameters were extracted from the nominal stress versus stretch curve^24^: the tangent modulus of the linear region, the heel region length, the failure stretch and the ultimate tensile stress.

### Development and evaluation of a cryopreservation protocol

In order to create a ready to use DHE bank, a cryopreservation method was validated. This procedure was carried out under sterile conditions. DHE were immersed in 100 mL RPMI (Invitrogen) with 10% dimethylsulfoxid (DMSO, WAK) and 0.8% human albumin. DHE and cryoprotectant solution were packed in capton-teflon bags (Hemofreeze, MedHem Science, NL). Samples were slowly cooled, at − 1 to − 2°C/min between 4 and − 10°C, − 2°C/min between − 10 and − 30°C, − 5°C/min between − 30 and − 50°C then − 10°C/min down to − 160°C, in Freezal (Air Liquide, France). DHE were cryopreserved for at least 3 weeks and thawed for further analyses. For thawing, they were kept at room temperature for 8 min then immersed in a water bath of 40°C until complete defrosting. The bags were then decontaminated, thawed DHE taken out and immersed in a series of decreasing concentrations of DMSO (8%, 4% and 2% respectively). They were then preserved in physiological serum at 4°C before further evaluation.

To evaluate the safety and incidence of the cryopreservation method on the quality of the esophagus, we analyzed and compared samples before cryopreservation (DHE stored in PBS at 4°C) and after thawing (cryo-DHE). For that, we assessed sterility, cytotoxicity, lymphocyte proliferation assay, histology with HES staining, SEM and biomechanical properties as previously described.

### Statistical analyses

Statistical analysis was performed with SPSS Statistics IBM Corp. (Released 2019. IBM SPSS Statistics for Macintosh, Version 26.0. Armonk, NY: IBM Corp). Data were expressed as proportions (%) and mean ± standard deviation. Univariate analysis was done with a Mann–Whitney U-test for the quantitative data (DNA, GAG and SDS quantification, as well as cytotoxicity analysis). Results of the elastin quantification were compared with an unpaired t-test, using Prism software (GraphPad).

Statistical analysis was performed using Jamovi for the biomechanical tests. Prior analysis, some values were removed: 2 heel-region for which the sample was already stretched initially, and 1 sample with abnormal linear part. T-tests were performed to compare the effect of the direction of the loading or the effect of the freezing on heel region length and tangent modulus.

### Ethics approval and consent to participate

Esophageal grafts were harvested from human deceased donors following a brain-death or Maastricht category III circulatory arrest. Non-opposition consent was obtained for research purposes. The graft retrieval authorization was granted by the "Agence de la Biomédecine", Grant Number PFS18-018 in Saint-Louis Hospital, Université Paris Cité, France.

## Results

### Treatment of the esophagi

A total of 20 esophagi were retrieved from human deceased donors and the decellularization protocol was applied to all of them between November 2019 and January 2022. One of them (DHE 5) was not exploitable because too short to be decellularized and was excluded from the analyses. Among them, 4 samples were treated with the ion exchange resin (n = 4) and the others with the activated charcoal cartridge (n = 15) to remove the residual SDS.

The cryopreservation protocol was evaluated in six DHE at the end of the decellularization process.

### Microbiological analysis

The decontamination step with ATB/ATM removed all bacterial contamination from all the samples. Only 3 samples (17%) were positive for fungi after the first decontamination step. However, at the end of the decellularization, every sample was negative for bacterial and fungal presence (Table 1). After cryopreservation, no contamination was reported for the different samples analyzed.

**Table 1:** Microbiological results of the samples at different times of the process. Abbreviations: GNB, Gram-Negative Bacilli; GPB, Gram-Positive Bacilli, GPC, Gram-Positive Cocci, Neg, negative for any culture, “n/a” indicates not applicable as these DHE were not cryopreserved.

Samples	Before decontamination with antibiotics and antimycotics	After 24h of decontamination with antibiotics and antimycotics	After decellularization	After cryopreservation and thawing
DHE01	Neg	Neg	Neg	n/a
DHE02	GPB/GNB	Neg	Neg	n/a
DHE03	GPB/GNB	Neg	Neg	n/a
DHE06	Candida albicans	Neg	Neg	n/a
DHE07	GPC/GPB/GNB	Candida albicans	Neg	n/a
DHE08	GPC/GPB/GNB	Neg	Neg	n/a
DHE09	GPB/GPC	Neg	Neg	n/a
DHE10	Candida tropicalis	Candida tropicalis	Neg	n/a
DHE18	GPB	Neg	Neg	n/a
DHE19	GPC/GPB/GNB	Neg	Neg	n/a
DHE20	Neg	Neg	Neg	n/a
DHE11	Candida glabrata	Candida glabrata	Neg	Neg
DHE12	GPC/GPB/GNB	Neg	Neg	Neg
DHE13	Neg	Neg	Neg	Neg
DHE14	GPB/GPC	Neg	Neg	Neg
DHE15	GPC/GPB/GNB	Neg	Neg	Neg
DHE16	GPB	Neg	n/a	Neg
DHE17	GPB	n/a	Neg	Neg

### Structural analysis

#### Macroscopic description

**Figure 2:**
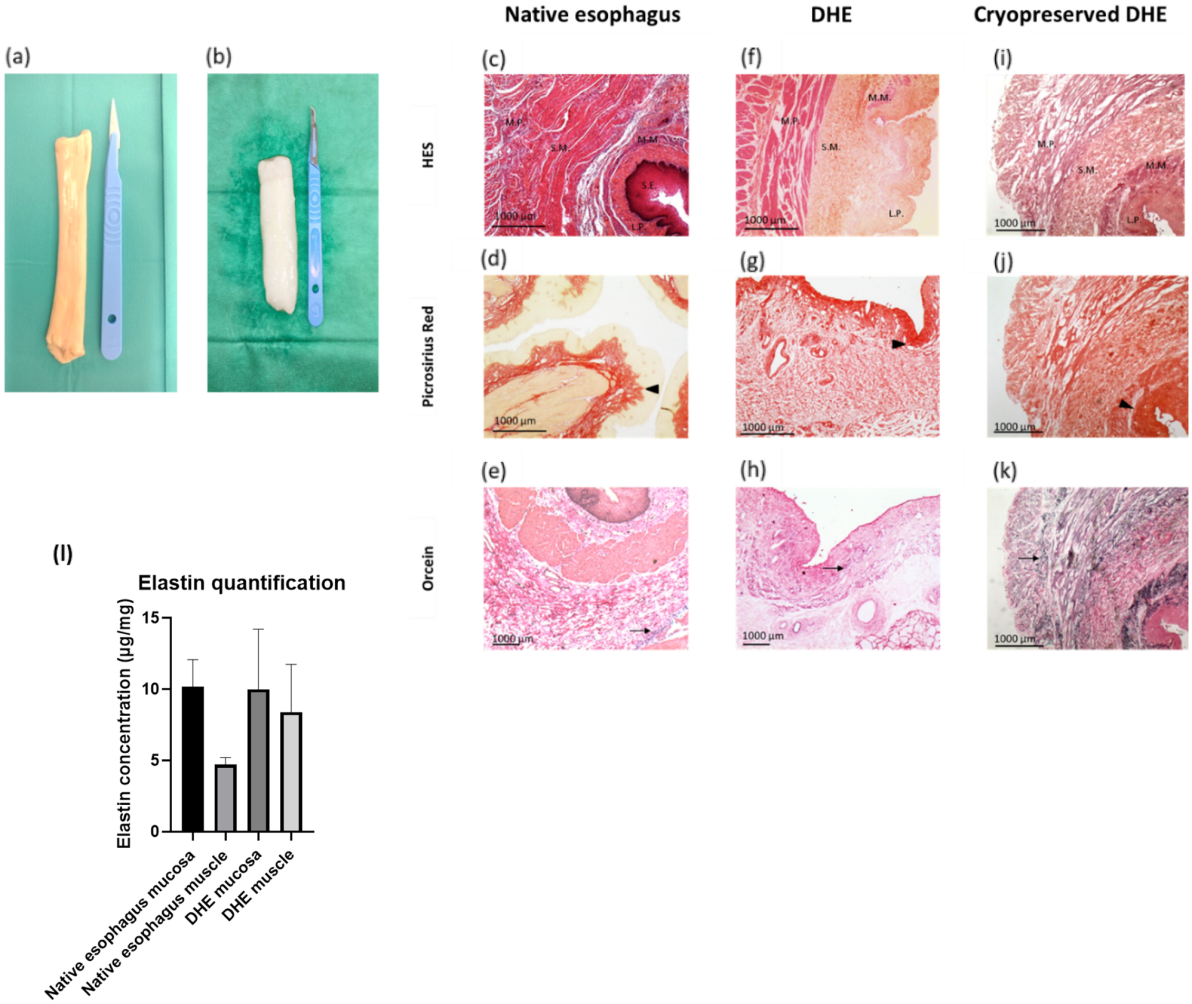
Macroscopic appearance of native (**a**) and decellularized esophagus (**b**). Histology of transversal sections of native (**c**–**e**) and decellularized esophagus (**f**–**h**) and cryopreserved/thawed (**i**–**k**) stained with: HES (**c,f**), picrosirius red (**d,g**) staining the collagen fibers (arrowhead), and orcein (**e,h**) staining the elastic fibers (arrow). *Abbreviations* L.P., lamina propria; M.M., muscularis mucosa; M.P., muscularis propria; S.E., superficial epithelium; S.M., submucosa; HES, hematoxylin eosin saffron. Elastin quantification with Fastin™—Elastin assay kit, after solubilization. Results are expressed in µg elastin/ mg wet tissue (l). No significant difference was observed between the samples.

The DHE preserved a tubular structure but showed a discoloration, as well as a parietal softening due to edema compared to the native esophagus (Fig. 2a,b). The mean weight of the native and decellularized samples was respectively 18.4 ± 4.5 g (n = 19) and 22.3 ± 6.6 g (n = 19, *p* < 0.001), representing a 20.5% mean weight gain during the process due to edema of the DHE. They also showed a permanent relaxation compared to the native esophagi, probably due to the removal of contractile muscle cells. After cryopreservation, the DHE had a comparable macroscopic aspect compared to unfrozen samples, with loss of edema.

#### Histological analysis

The general structure of the esophagus was preserved after decellularization and four layers well identified by microscopic examination. HES staining demonstrated the efficiency of the decellularization with a complete removal of cells and their nuclei in all the layers of the DHE (Fig. 2c,f). Picrosirius red staining showed that despite some loss and disorganization of collagen fibers, the densest zone remained in the lamina propria (Fig. 2d,g). Orcein staining showed a conservation of elastic fibers after decellularization (Fig. 2e,h). DHE showed an important edema compared to the native esophagus, confirming the macroscopic evaluation. After cryopreservation, samples showed similar results with no major damage to the ECM (Fig. 2i–k).

Elastin quantification showed that in the native esophagus, the mucosa contains 10.21 ± 1.88 µg/mg and the muscle contains 4.72 ± 0.48 µg/mg elastin. The concentration found in DHE mucosa and muscle were 9.99 ± 4.23 and 8.39 ± 3.35 µg/mg respectively. No statistically significant difference was observed between the different samples. Elastin concentrations in the mucosa were higher compared to the muscle, especially in the native esophagus. However, no significant difference was observed between the different samples (Fig. 2l). These results show that the decellularization protocol preserved elastin in the ECM, confirming the results of histology.

#### SEM analysis

**Figure 3:**
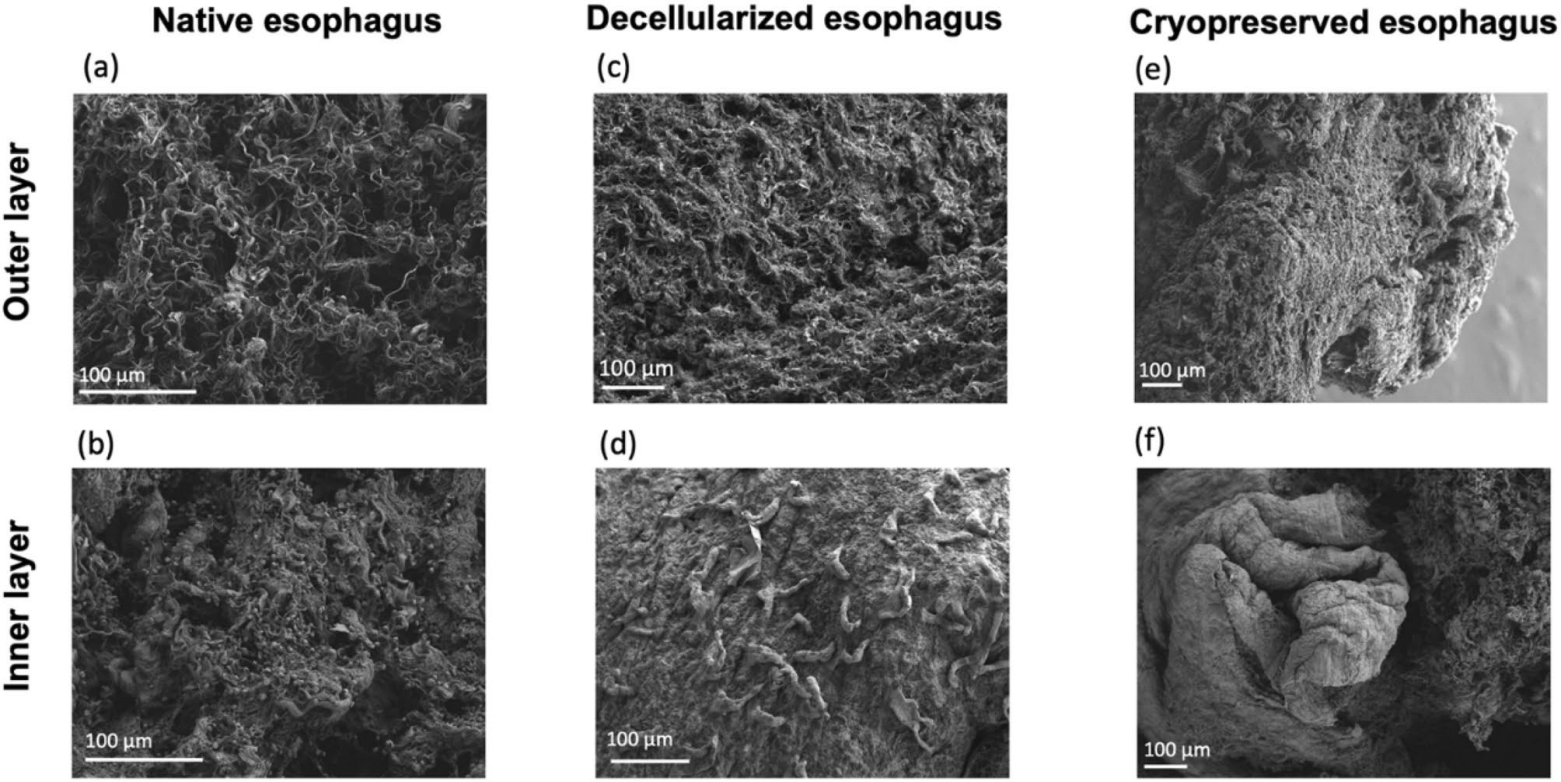
Scanning Electron Microscopy of native (**a**,**b**), decellularized (**c**,**d**) and cryopreserved esophagus (**e**,**f**). The absence of cell is confirmed after decellularization (**b**–**f**). The structure with collagen fibers is preserved after decellularization and after thawing.

The efficiency of the decellularization was confirmed by SEM analysis. The decellularized samples had no visible cells compared to the native esophagus and the structure of collagen fibers seemed to be preserved (Fig. 3a,b). In the DHE, the lumen composed of the basement membrane presented a dense and compact composition of collagen whereas the outer layer showed a looser zone of fibrillar collagen (Fig. 3c,d). Cryopreservation allowed to conserve the microstructure of the DHE and no major damage was visible (Fig. 3e,f).

### DNA extraction and quantification

**Figure 4:**
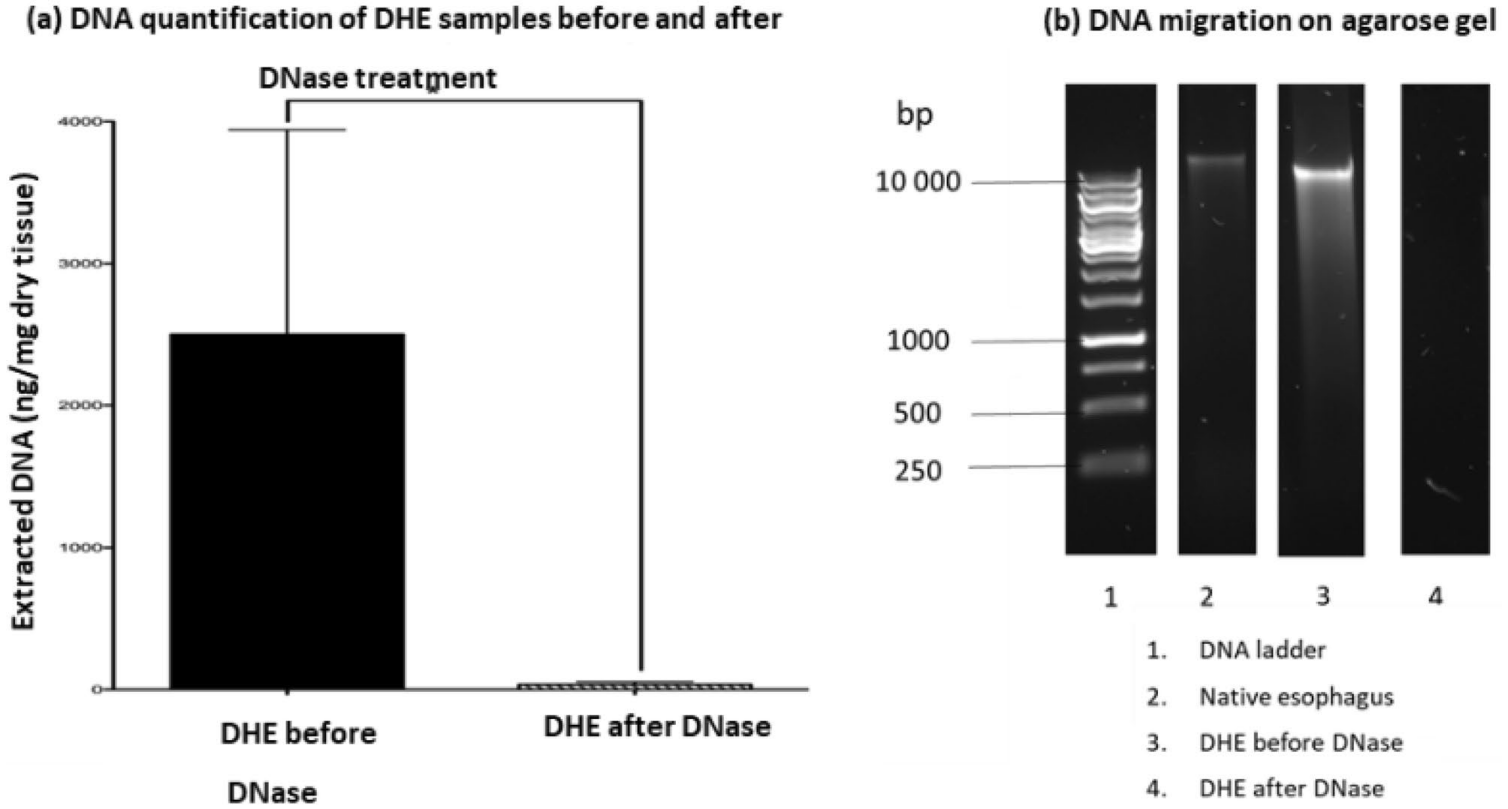
DNA quantification before and after DNase incubation measured by Nanodrop (**a**). Extracted DNA fragments size measured by migration on agarose gel read by Chemidoc.

The residual DNA concentrations were of 2501 ± 1444 ng/mg of dry tissue (*n* = 4) for DHE before DNase treatment and of 35.5 ± 19.5 ng/mg after DNase (*n* = 7, *p* < 0.01). Before DNase treatment, the residual DNA fragments length was over 1000 bp, similar to the native esophagus. After DNase, residual DNA was not detected (Fig. 4a,b).

### GAGs analysis

**Figure 5:**
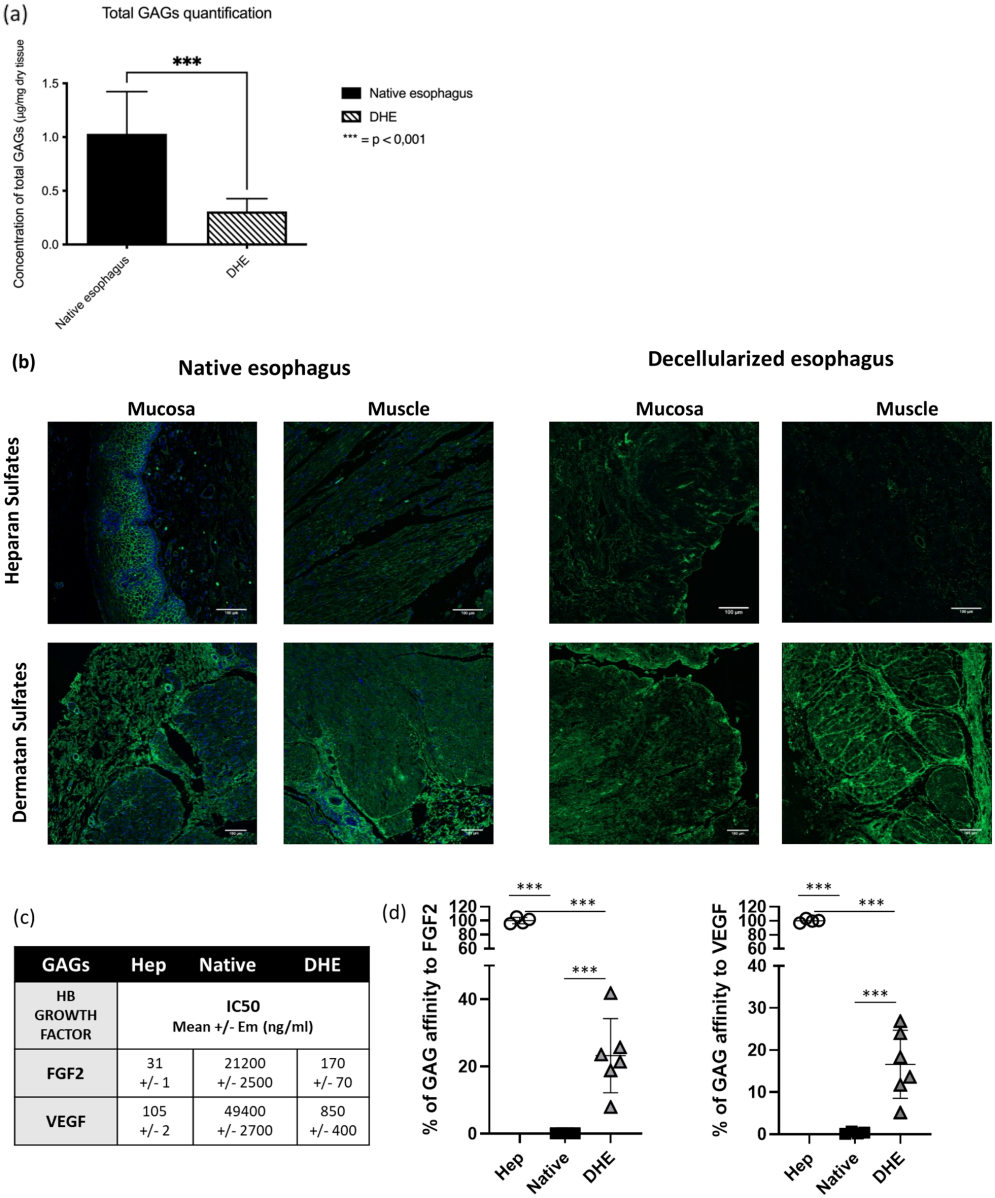
GAG Characterizations: (**a**) Total GAGs quantification (µg/mg of dry tissue) before (Native) and after (DHE) decellularization of esophagus. (**b**) Slices of native and decellularized esophagi immunostained of HS with EV3CV3 to observe the remaining HS, DS with LKN1 G3998/EP1143 for DS and DAPI staining for cell nuclei (**b**). White bars indicate 100µm. (**c**) IC50 values (ng/ml) of tested GAG (Heparin, native or HEB) to compete for 50% of FGF2 or VEGF binding to Heparin coated on Elisa plates in competitive binding test. (**d**) % of GAG affinity to HB growth factor as compared to Heparin IC50 as 100%. Statistical analysis according to Shapiro and Mann–Whitney tests. ***p < 0.001.

Total GAGs concentrations for the native esophagi were 1.03 ± 0.2 μg/mg dry tissue (*n* = 4) and for DHE 0.31 ± 0.04 μg/mg dry tissue (*n* = 8*, p* < 0.01) representing a 70% total GAGs loss during the decellularization process. (Fig. 5a).

Immunostaining of HS with EV3C3V showed that in the native esophagus, these molecules were mainly present in the mucosa between epithelial cells, and to a lesser extent in the submucosa and the muscle layer. In the DHE, a major loss of these HS was observed with the elimination of the epithelium but they could still be detected in the basement membrane in the lumen and around blood vessels in the muscle layer. As for dermatan sulfates which are part of CS, staining with LKN1 showed a more uniform distribution of these species in the different layers of the native esophagus and they were strongly preserved in DHE (Fig. 5b).

GAGs capacities to regulate cells functions is largely related to their specific abilities to bind to HBPs. Binding affinity of native and DHE GAGs toward HBP such as FGF-2 and VEGF, were evaluated according to Elisa binding competitive assays that permit to measure IC50 doses of GAG necessary to compete for 50% of HBP binding to Heparin. Native GAGs were able to bind to FGF2 and VEGF and compete for Heparin with respective IC50 (21,200 and 49,400 ng/ml) that were 680 times and 470 times higher than Heparin IC50 as positive control, (31 and 105 ng/ml for FGF2 and VEGF respectively). DHE GAGs were able to bind strongly to FGF2 and VEGF with 170 ± 70 and 850 ± 400 ng/ml IC50 respective values, on the same range than Heparin IC50 (31 and 105 ng/ml respectively). As compared to 100% Heparin affinity to FGF2 and VEGF, DHE GAGs have 23 ± 7% and 17 ± 6% binding affinity to FGF2 and VEGF. This suggest that DHE matrix are enriched in functional GAGs (Fig. 5c,d).

### In vitro biocompatibility assays

#### Cytotoxicity analyses on Balb/3T3 cells

The percentage of viable Balb/3T3 cells in the extraction supernatant of the DHE was 94.8 ± 2.0% (*n* = 12), comparable to the control conditions with a viability of 90.9 ± 5.5% (*n* = 11, *p* = 0*.*04). These results demonstrated the absence of cytotoxicity induced by the DHE.

**Figure 6:**
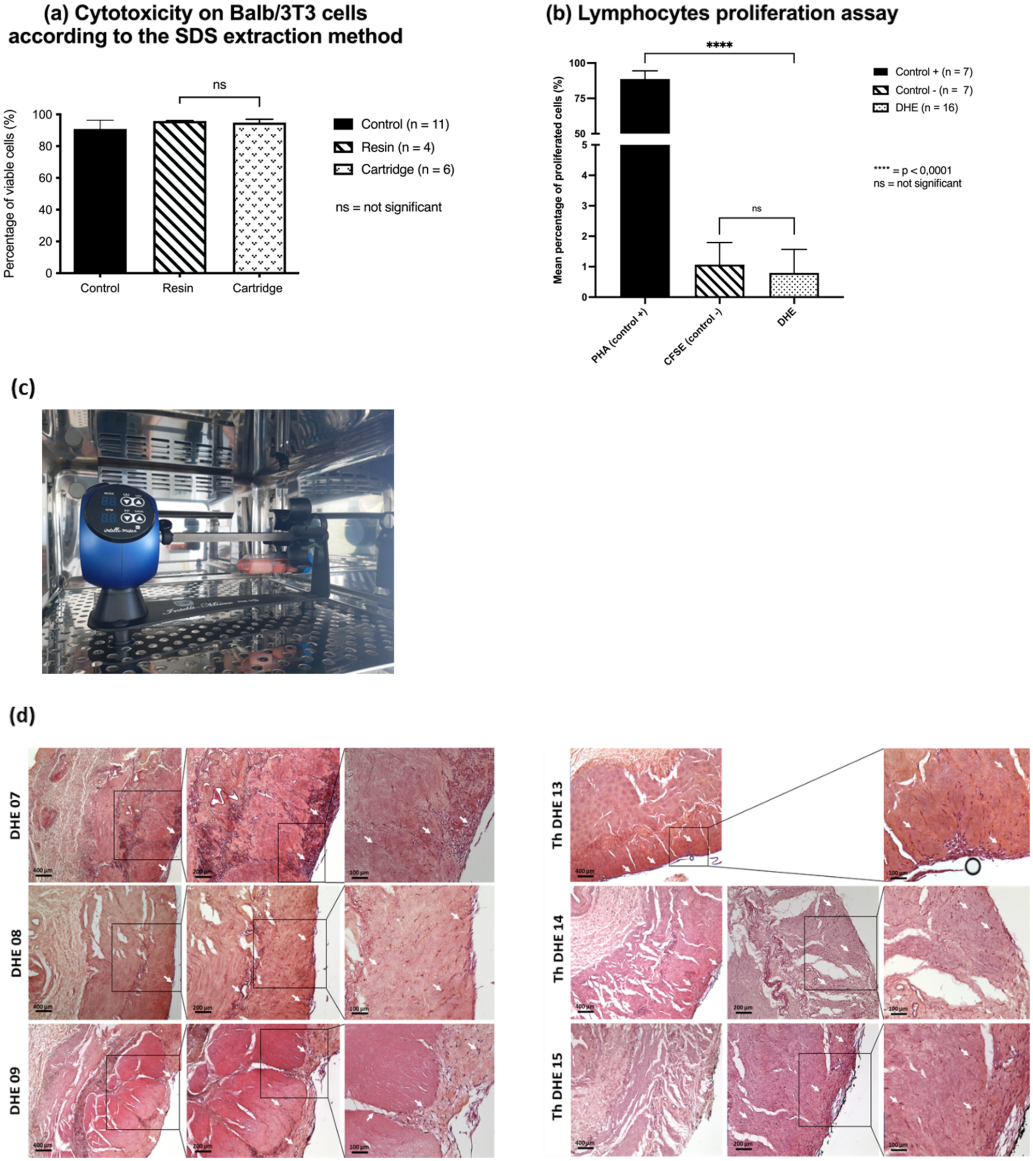
Cytotoxicity of DHE on Balb/3T3 cells after decellularization. DHE was not cytotoxic with a percentage of viable cells superior to the control group. The SDS extraction with activated charcoal cartridge was efficient and did not induce cytotoxicity compared to the ion exchanger resin (**a**). DHE did not induce lymphocytes proliferation (**b**) compared to the positive control with PHA. Samples of DHE on the rotating agitator seeded with MSCs during 14 days (**c**). Immunohistochemistry with HES coloration of DHE samples after cell seeding with hMSCs at 3 magnifications (× 2.5, × 5 and × 10). Cells have attached (white arrows) on the mucosal layer and mainly on the external layer. Infiltration of cells was observed from the outer layer (**d**). Th DHE: Thawed DHE.

The percentage of viable cells were similar between DHE treated by the ion-exchange resin and by the charcoal cartridge with 95.8 ± 0.33% (*n* = 4) and 95.2 ± 1.7% (*n* = 6*, p* = 0*.*39) viable cells respectively (Fig. 6a).

#### Lymphocyte proliferation assay

The positive control of lymphocytes treated with PHA showed a strong proliferation (88.7 ± 5.78%) whereas the negative untreated control showed a very low proliferation (1.07 ± 0.72%). As for lymphocytes that were in contact with DHE, the proliferation percentages were 0.76 ± 0.77% (n = 16, Fig. 6b). These results show that the DHE did not induce in-vitro the activation of lymphocytes.

#### Cell seeding with MSCs

After 14 days of culture in a rotating agitator system (Fig. 6c), attachment, colonization and proliferation of hMSCs were observed in the DHE before or after cryopreservation. Cell attachment was more visible on the outer layer, in direct contact with cell suspension than the inner mucosal layer (Fig. 6d). Cell infiltration was also observed mainly from the outer layer. MTS assay confirmed the present of living and functional hMSC on the DHE (Figure S1a). hMSC quantification in DHE were before and after cryopreservation 2.9 ± 1.2 and 2.1 ± 0.5 cells/mm3 respectively (Figure S1b,c). These results confirmed the migration of primary cells on DHE with a survival before and after cryopreservation.

### Residual SDS quantification

**Figure 7:**
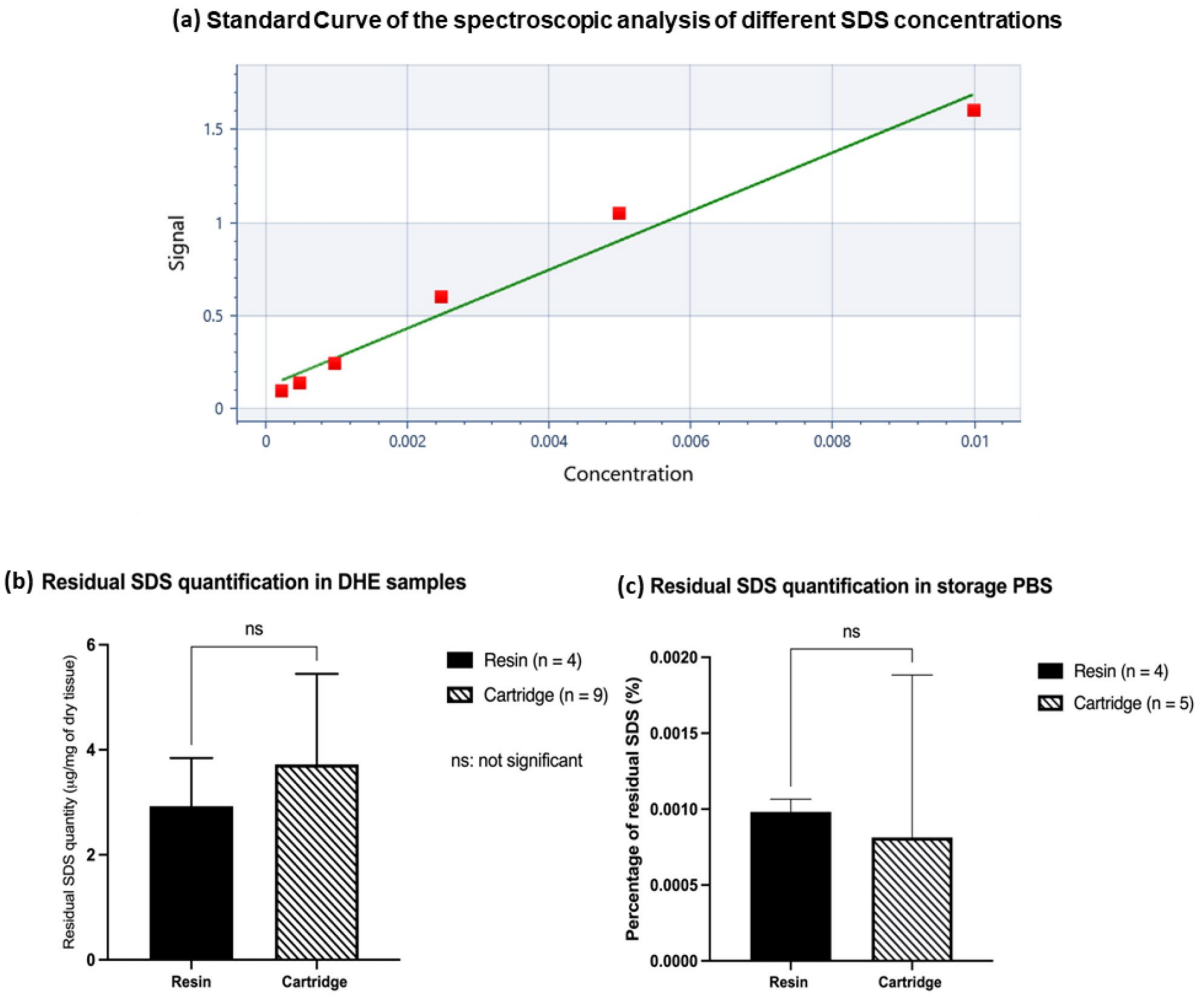
Standard Curve for the spectroscopic analysis of different SDS concentrations (**a**). Quantification of residual SDS in the DHE samples after dehydration and extraction (**b**) and in the storage PBS (**c**) obtained by spectroscopic analysis.

The first step consisted of validating the detection method and limit of the colorimetric dosage. A linear standard curve was first validated using a range of known concentrations of SDS and a blank consisting of PBS (Fig. 7a). Blank values obtained were typically about 0.0993 ± 0.0027%, and we thus estimate the detection limit of this procedure for SDS in phosphate buffer, to be about 0,000,027%, which correspond to 0.9 µM or 0.27 ng/µL.

The dosage of SDS showed that it was strongly eliminated by both the ion exchange resin and the charcoal cartridge. The mean concentration for samples treated with ion exchange resin was 2.9 ± 0.9 µg/mg of dry tissue (n = 4) with no significant difference from those treated with activated charcoal was 3.7 ± 1.7 µg/mg of dry tissue (n = 9, *p* = 0*.*4*,* Fig. 7b). As for the SDS quantities in the storage PBS, there was no difference between the two types of SDS extraction on the residual quantification. DHE treated with the ion-exchange resin showed 0.0009820 ± 8.348.10^−5^% of residual SDS (n = 4) and the DHE treated with the cartridge showed 0.0008138 ± 0.001070% of residual SDS (n = 5, *p* = 0.76, Fig. 7c).

### Biomechanical properties after cryopreservation

**Figure 8:**
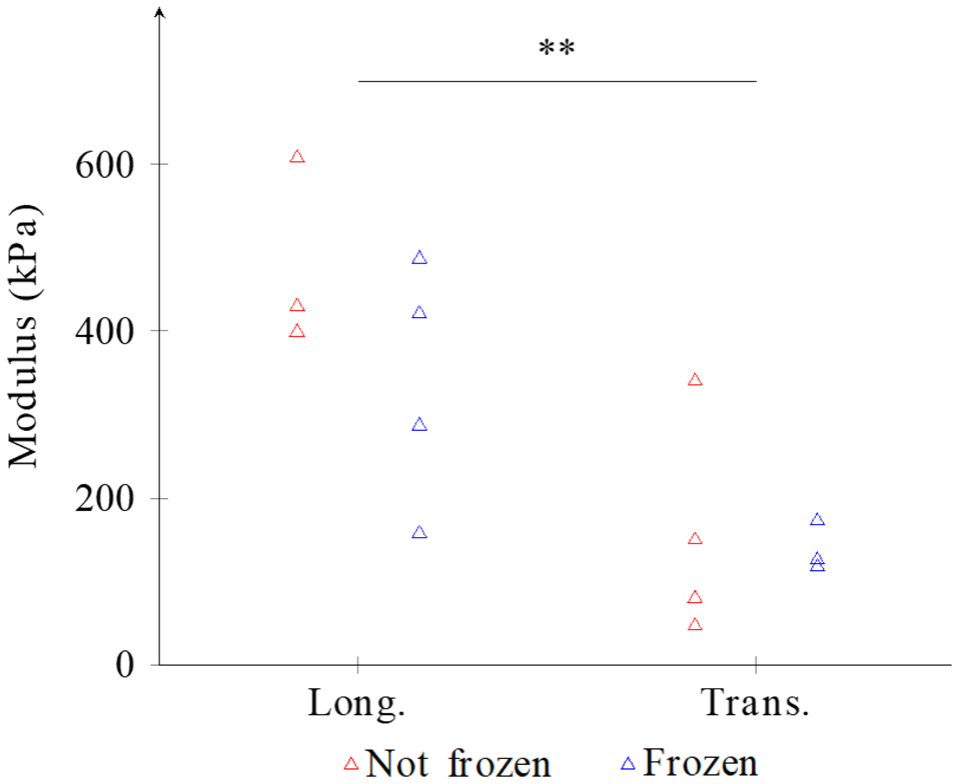
Tangent modulus for frozen and not-frozen samples, in the longitudinal (left) and transverse (right) direction. Bars represent the mean. Points are the individual data. Abbreviations: Long. Longitudinal, Trans. Transversal.

The comparison of the tangent moduli of the different samples tested showed a statistical difference only between the longitudinal and transverse directions. Longitudinal samples were much stiffer compared to transverse ones (Fig. 8).

No significant difference was observed between cryopreserved and not-cryopreserved samples. Analysis of the length of the heel region led to the same conclusion. These results show that cryopreservation does not cause any major degradation to the DHE.

## Discussion

Tissue engineering is a promising alternative for circumferential esophageal replacement, preserving healthy intra-abdominal organs and the healthy part of the esophagus, unlike current methods of esophageal reconstruction as gastroplasty or coloplasty^25^. We have previously shown that the use of a decellularized porcine esophagus (DPE) for a full thickness circumferential replacement allows the recovery of nutritional autonomy in pigs, if the graft area is temporary covered by an esophageal stent. In vivo tissue remodeling toward an esophageal phenotype was observed after a few weeks. We have also shown that seeding the DPE with autologous MSCs has no influence on clinical and histological outcomes. Our findings support the idea that the ECM of the esophagus per se has the biochemical and biomechanical properties that enhance a specific tissue remodeling.

An efficient esophageal decellularization results in a complete removal of cells, without compromising the architectural and biochemical properties of the ECM. Removal of all DNA fragments is paramount to reducing acute and chronic pro-inflammatory reactions which lead to fibrotic reactions that compromise tissue remodeling^23^. Several teams have proposed different physical, chemical or enzymatic decellularization methods, on animal models of different sizes^12,15,26–28^. However, despite promising results, these protocols were used for research purposes only, often with limited in vitro and in vivo characterization. In order to facilitate a transition towards a clinical use, we previously demonstrated the proof of concept of a “clinical grade” porcine decellularized esophagus (DPE), using a bioreactor and reagents with medical application approval. Furthermore, we showed the lack of cytotoxicity of the DPE using European ISO 10993-5-2009 guidelines for in vitro assays. However, the use of DPE in humans has certain limits due to the presence of xenoantigens such as alpha-galactosyl epitopes that are immediately bound to natural antibodies in human^17^ can lead to hyperacute rejection^29^. Another limit of graft of porcine tissue would be the transmission of potential porcine viruses^30^. We therefore decided to adapt our previous decellularization protocol to human esophagi.

Human esophageal retrieval was performed easily with the same method used for porcine esophageal retrieval. Transhiatal retrieval was performed in the absence of pulmonary or cardiac harvesting. In these cases, a transhiatal approach showed no major difficulty and allowed esophageal retrieval with a sufficient length for insertion in the bioreactor.

Transposition of the decellularization protocol developed in pigs led to similar results for human esophagi. At the end of the decellularization process, all DHE were sterile. A number of samples (21%) were positive for fungal culture after the decontamination step with ATB/ATM treatment. The five most common pathogens of esophageal candidiasis in human were found^31^. The positivity of these samples could be explained by an amphotericin B resistance although not frequent^32^ in human or a low dosage of antimycotic. However, SDS treatment and multiple washing steps under sterile conditions got rid of these pathogens by the end of the decellularization process. These mycotic initial contaminations confirm the choice of an esophageal retrieval at the end of the organ procurement procedure in order to avoid contamination of other organs.

Macroscopically, the DHE presented a discoloration compared to the native esophagi; the tubular shape of the scaffold, was preserved, even though parietal edema was observed inducing a weight gain of 20.5% compared to the native esophagus. It is possible that this edema is due to the increased porosity of the layers of the ECM secondary to its decellularization and to the use of water solution with low osmolarity. This increase in porosity of a decellularized esophagus has already been shown but it did not major the anastomotic leak rate after esophageal replacement in a porcine model^33^. It could even be associated with a better cell colonization of the substitute^34^.

Microscopically, histology and SEM studies showed that the cells were fully removed, while preserving some general structure and ECM components. Furthermore, DAPI staining and DNA quantification showed that DNA and cell nuclei were strongly eliminated, respecting the recommended limit of 50 ng/mg of tissue and cell fragments of smaller than 200 bp. These results showed that the decellularization protocol previously developed on porcine esophagi was efficient on the human esophagi, using the same reagents and treatment durations.

The quantification of GAGs showed that like for the DPE, there was a major loss of these molecules in DHE as well. In the native human esophagi, HS were mainly found in the epithelium, and to a lesser extent around blood vessels. After decellularization and the elimination of the epithelium, a loss of these molecules was observed. On the other hand, DS had a more ubiquitous expression both in the mucosa and the muscularis and were well preserved after decellularization. Moreover, in human esophagi ECM analysis, we demonstrated that esophageal GAGs are able to interact with growth factors such as FGF2 and VEGF. These functional properties are not only maintained in DHE but also enriched.

One of the main issues for a future clinical use and the success of recolonization of the DHE by cells of the recipient was the elimination of potential toxic agents used for the decellularization process. SDS is a powerful detergent inducing cell lysis and preventing cell attachment^35^. Due to its protein-binding properties^36^, long and efficient rinsing steps were required to eliminate the remaining traces^37^. In the literature, different protocols have been studied to separate SDS from complex solutions, including column chromatography using the ion-retardation resin AG11A8^38^ or nonionic macroporous Amberlite XAD resins^39^. In our previous study, we used Amberlite^®^ XAD16N (Sigma, France) resin to remove the residual SDS from the DPE^15^. The resin efficiently removed the residual detergent from the DHE as well, when the same protocol of 72 h was applied. However, due to the lack of Good Manufacturing Practice (GMP) qualification, the resin could not be used for a clinical production. We therefore evaluated the use of a clinically approved product, the activated charcoal cartridge. This product is routinely used in human for hemofiltration in intensive care units^40^ and can also adsorb SDS from complex protein solutions^41^. For the residual SDS to be eliminated from the DHE, it first has to be efficiently released from it. SDS is a detergent that precipitates at temperatures below 15°°C and its solubility increases with temperature^42^. Therefore, we chose to perform SDS removal at 30°°C, in order to increase its solubility. However, higher temperatures were avoided in order to prevent further protein denaturation. Instead, we chose a detoxification period of 72 h. We did not observe any visible damage with both resin and activated charcoal cartridge methods.

The DHE is intended to be grafted in patients, at a body temperature of 37°°C. This was our upper limit for choosing the different treatment temperatures at the different stages of the protocol. When this new method of SDS extraction was applied to DHE, no cytotoxicity was observed on Balb/3T3 cells. Furthermore, SDS quantification with the colorimetric method showed that this detergent was removed both by resin and activated charcoal, confirming the efficiency of the new method. Cebotari et al*.*^43^ showed that under 50 mg/L (or 0.005%) of residual SDS in the washing liquid after decellularization of cardiac valves, no cytotoxicity was observed on human endothelial cells. The concentrations detected in the PBS used for DHE preservation were lower than this threshold, confirming the efficacy of our detergent removal. As for our standardized method of SDS quantification (mg SDS/mg of dry DHE mass), we did not find any threshold reference in the literature. However, our indirect method of cytotoxicity assay on Balb/3T3 cells and the seeding of the DHE with MSCs proved that the DHE allowed colonization and proliferation of cells and did not induce cell mortality. Furthermore, DHE did not induce a proliferation of lymphocytes in vitro, showing that it does not induce an acute activation of these cells.

Finally, in order to create a ready-to-use DHE bank, a cryopreservation method was developed, with solutions and instruments used by human tissue banks. This cryopreservation protocol was adapted from a previous protocol used for arterial allograft replacement for aortic infections^44^.

Cryopreserved and thawed DHE did not present any macroscopic visible damage and showed a loss of edema, compared to freshly decellularized samples. Microscopically, histology and SEM results showed that the different tissue layers and ECM fibers were undamaged after cryopreservation. These results were further confirmed by biomechanical assays, showing similar tensile properties between cryopreserved and unfrozen samples adding to the reliability of our cryopreservation process.

## Conclusion

To conclude, to our knowledge, this study represents the first report of a clinical grade human esophageal decellularization process, paving the path to a near future clinical application in patients. We hereby present the largest series of clinical grade DHE development and characterization. In pursuit of the development of a clinical trial, the process of DHE production was transferred to the Human Tissue Bank of St. Louis hospital for clinical grade production. Thanks to this work, our team currently received a funding from the French ministry of health for the development of a multicentric phase I/II clinical trial of circumferential esophageal replacement by our DHE.

### Supplementary Information

Supplementary Figures.

## Data Availability

The datasets used and/or analyzed during the current study available from the corresponding authors on reasonable request.

## Abbreviations

ATB: Antibiotic
ATM: Antimycotic
BCS: Bovine calf serum
CFSE: Carboxyfluorescein succinimidyl ester fluorescent
CS: Chondroitin sulfate
DAPI: 4′, 6‐Diamidino‐2‐phenylindole
DHE: Decellularized human esophagus
DMSO: Dimethyl sulfoxide
DPE: Decellularized porcine esophagus
DS: Dermatan sulfate
ECM: Extracellular matrix
EDTA: Ethylendiaminetetraacetic acid
FBS: Fetal bovine serum
GAG: Glycosaminoglycan
HBP: Heparin binding protein
HES: Haematoxylin, eosin, saffron
hPBMC: Human peripheral blood mononuclear cells
HS: Heparan sulfate
MSC: Mesenchymal stromal cell
PD: Percentage of division
PHA-L: Phytohemagglutinin‐L
RPMI: Roswell Park Memorial Institute glutamax
SDS: Sodium dodecyl sulfate
TE: Tissue engineering
