# Supplementary material for: Development and qualification of clinical grade decellularized and cryopreserved human esophagi
Source: Sci Rep. 2023 Oct 25;13:18283. doi: 10.1038/s41598-023-45610-5 (PMC10600094; doi:10.1038/s41598-023-45610-5)
Supplement: Supplementary file 1 — Supplementary Figures. [file 41598_2023_45610_MOESM1_ESM.docx]

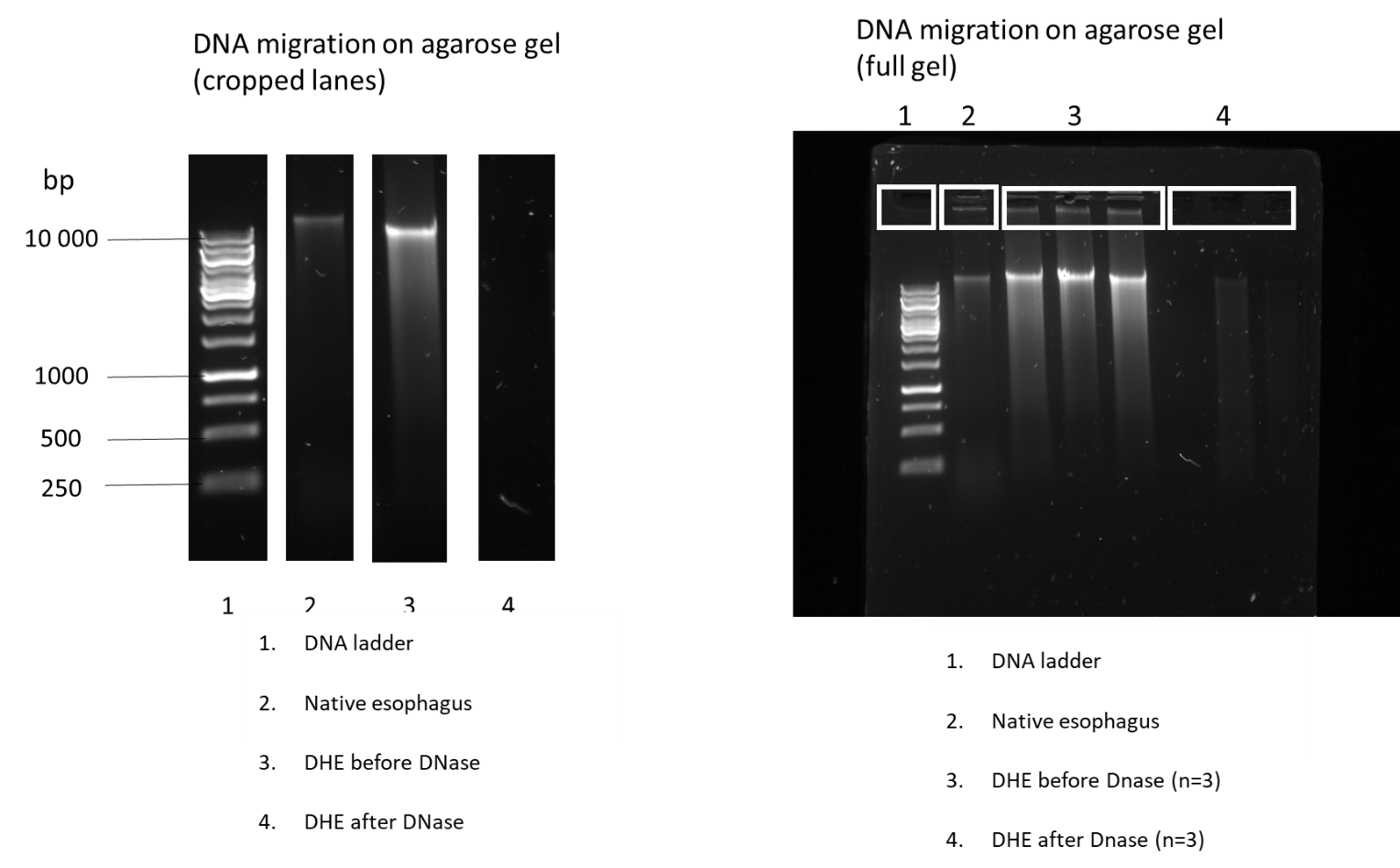


DNA migration on agarose gel: cropped gel used for main figure 4b (left) and full length gel used to create the figure (right).


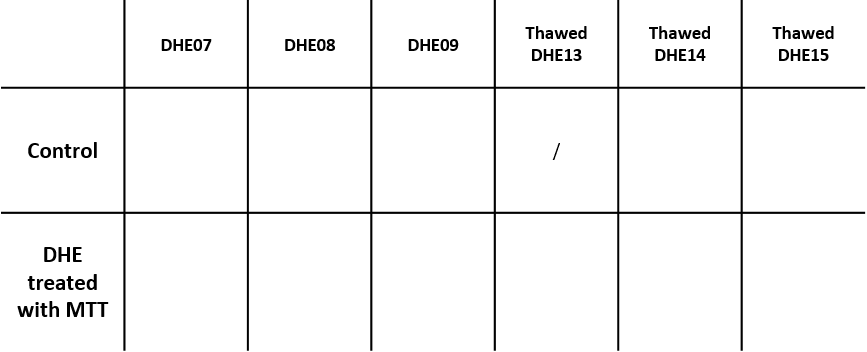

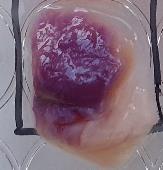

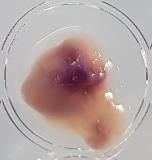

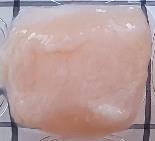

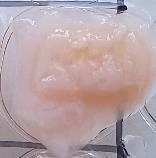

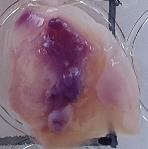

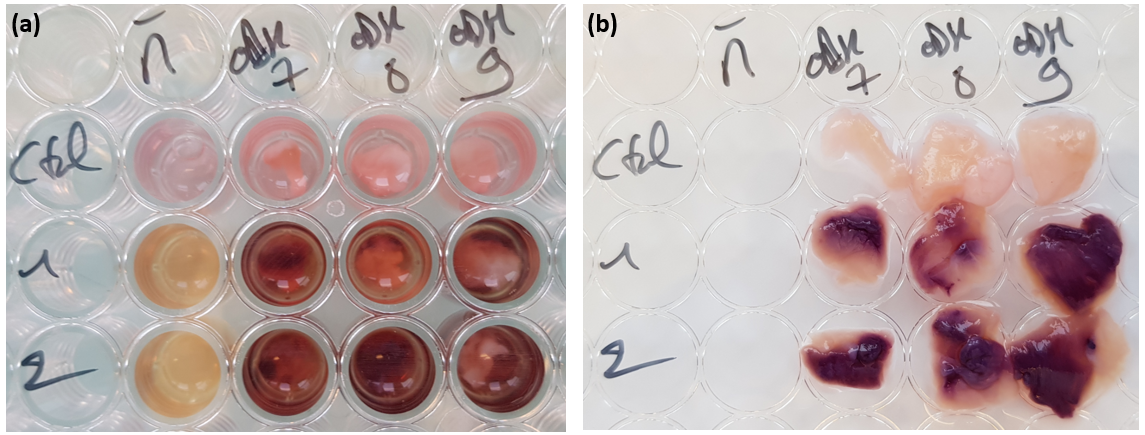

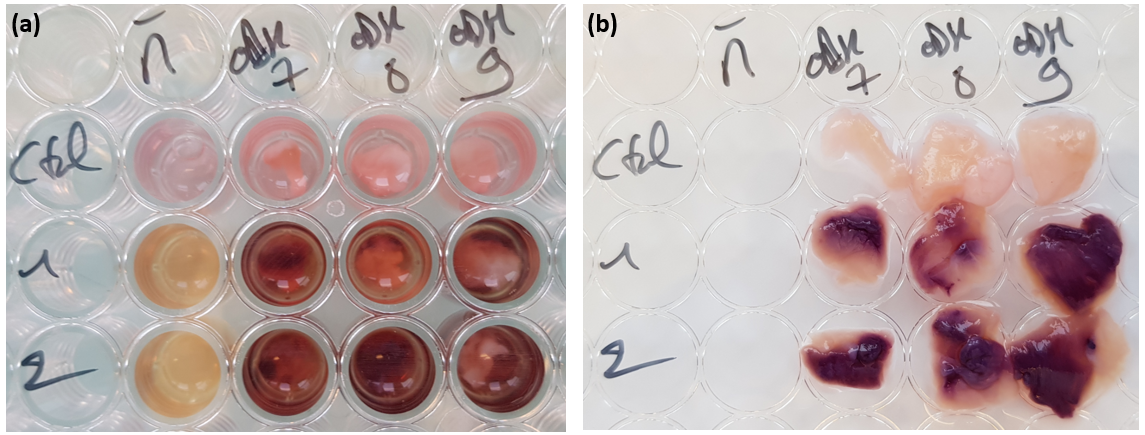

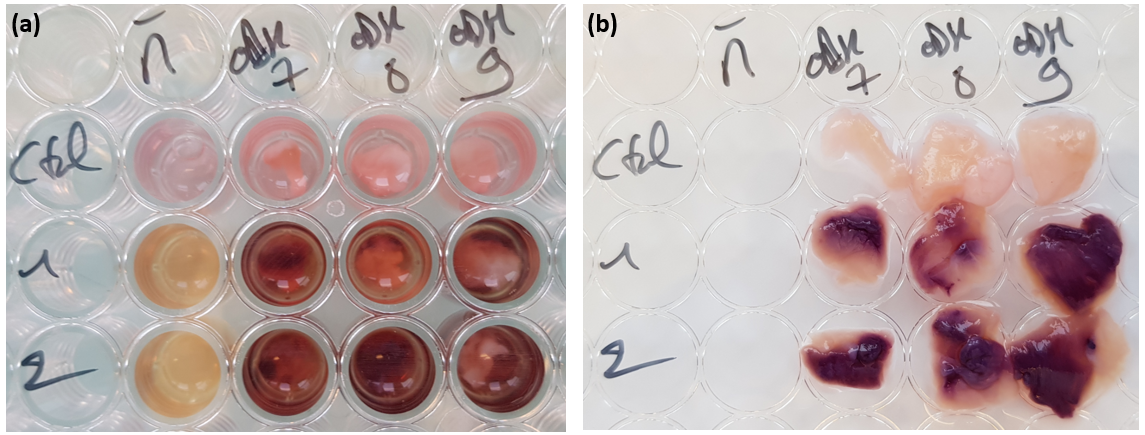

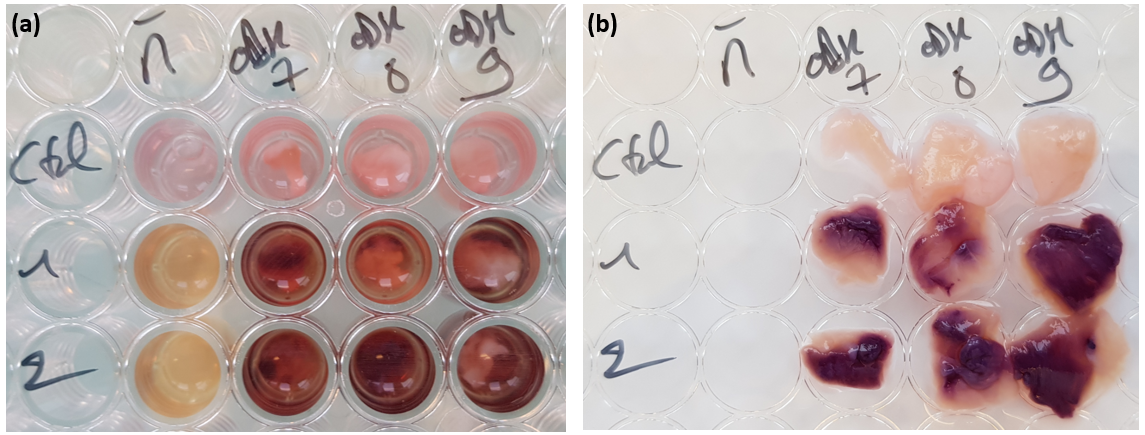

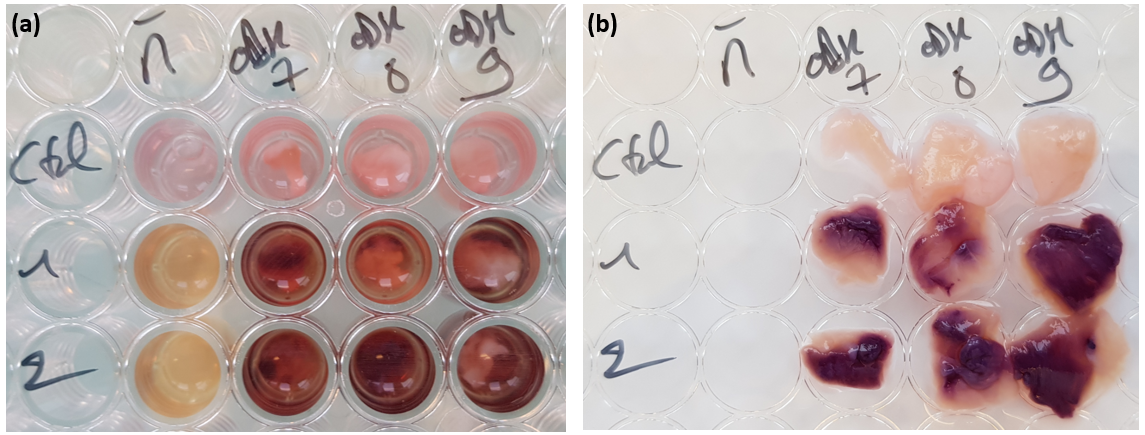

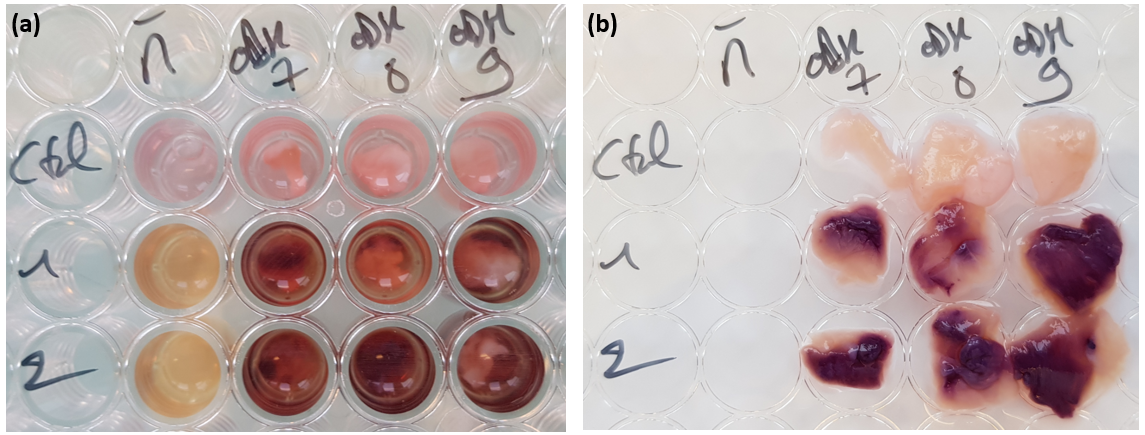

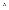

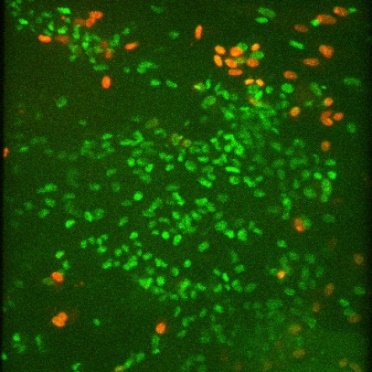

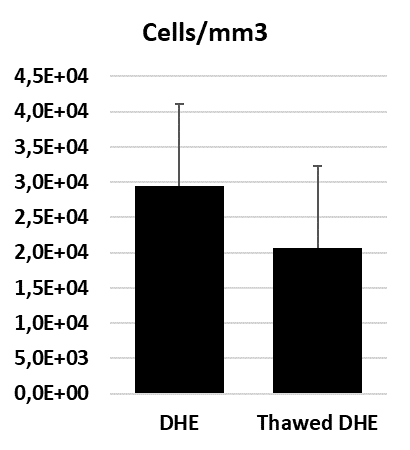


**Figure S1:** Biocompatibility with hMSC a) Qualitative evaluation of metabolic activity by colorimetric test (MTS) of DHE07, 08, 09 and thawed DHE13, 14 and 15 after 14 days of culture with hMSC. The control corresponds to DHE without MTS. DHE and thawed DHE showed a purple color if living and functional hMSCs are present. Real size pictures. b) Pictures of DHE after propidium iodide (Red) and Hoescht (green) labeling used to quantify viable hMSC in seeded DHE ; scale bar 50 µm. c) Cell quantification on 3 samples before and after cryopreservation with respectively 2,9±1,2 and 2,1±0,5 cells/mm3.

**(a)**

**(b)**


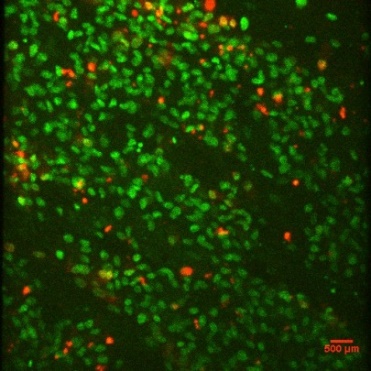


**50 µm**

**(c)**
